# Supplementary material for: The sound of a Martian dust devil
Source: Nat Commun. 2022 Dec 13;13:7505. doi: 10.1038/s41467-022-35100-z (PMC9747922; doi:10.1038/s41467-022-35100-z)
Supplement: Supplementary file 1 — Supplementary Information [file 41467_2022_35100_MOESM1_ESM.pdf]

# - SUPPLEMENTARY INFORMATION -

The Sound of a Martian Dust Devil

N. Murdoch[1*], A. E. Stott[1], M. Gillier[1], R. Hueso[2], M. Lemmon[3], G. Martinez[4,5], V. Apéstigue[6], D. Toledo[6], R. D. Lorenz[7], B. Chide[8], A. Munguira[2], A. Sánchez-Lavega[2], A. Vicente-Retortillo[9], C. E. Newman[10], S. Maurice[11], M. de la Torre Juárez[12], T. Bertrand[13], D. Banfield[14,15], S. Navarro[9], M. Marin[9], J. Torres[9], J. Gomez-Elvira[6], X. Jacob[16], A. Cadu[1], A. Sournac[1], J. A. Rodriguez-Manfredi[9], R. C. Wiens[17] and D. Mimoun[1]

*Corresponding author. Email: naomi.murdoch@isae-supaero.fr

[1]Institut Supérieur de l'Aéronautique et de l'Espace (ISAE-SUPAERO), Université de Toulouse, Toulouse, France
[2]Física Aplicada, Escuela de Ingeniería de Bilbao, Universidad del País Vasco (UPV/EHU), Bilbao, Spain
[3]Space Science Institute, Boulder, CO 80301, USA.
[4]Lunar and Planetary Institute, Universities Space Research Association, Houston, TX, USA,
[5]Department of Climate and Space Sciences and Engineering, University of Michigan, Ann Arbor, MI, USA
[6]Instituto Nacional de Técnica Aeroespacial, Spain
[7]Space Exploration Sector, Johns Hopkins Applied Physics Laboratory, Laurel, Maryland, USA
[8]Space and Planetary Exploration Team, Los Alamos National Laboratory, Los Alamos, New Mexico, USA
[9]Centro de Astrobiología (INTA-CSIC), Madrid, Spain
[10]Aeolis Research, Chandler, Arizona, USA
[11]Institut de Recherche en Astrophysique et Planétologie, Université de Toulouse 3 Paul Sabatier, CNRS, CNES, Toulouse, France
[12]Jet Propulsion Laboratory, California Institute of Technology, Pasadena, California, USA
[13] Laboratoire d'Etudes Spatiales et d'Instrumentation en Astrophysique (LESIA), Observatoire de Paris, Université PSL, CNRS, Sorbonne Université, Univ. Paris Diderot, Sorbonne Paris Cité, 5 place Jules Janssen, 92195 Meudon, France
[14]Cornell University, Ithaca, NY, USA
[15]NASA AMES Research Center, Moffett Field, CA, USA.
[16]Institut de Mécanique des Fluides, Université de Toulouse III Paul Sabatier, INP, CNRS, Toulouse, France
[17]Earth, Atmospheric, and Planetary Sciences, Purdue University, West Lafayette, Indiana, USA

# - SUPPLEMENTARY FIGURES -

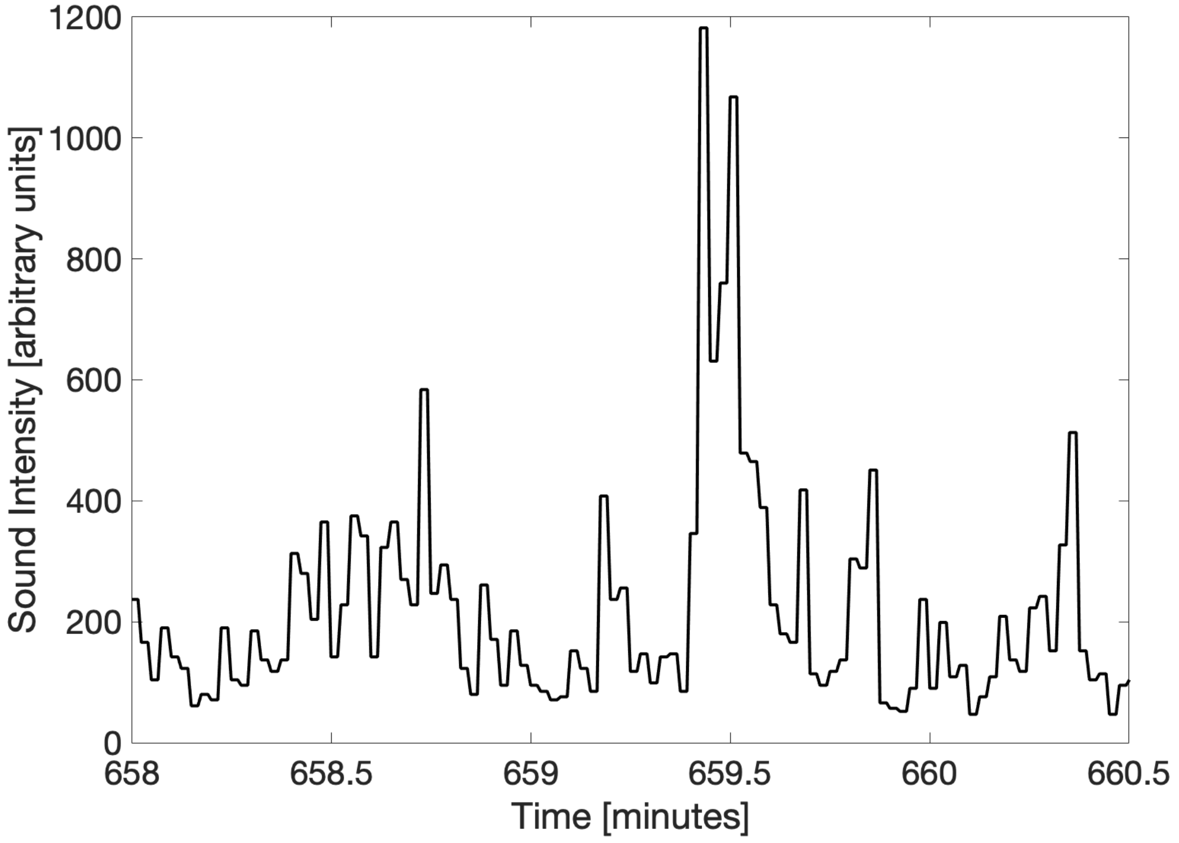

Supplementary Fig. 1 | Sound recording of a terrestrial vortex. The sound intensity recorded during a vortex encounter at Goldstone Dry Lake, California USA in June 2014[1]. Notice the saddle-shaped peak, with the two local maxima in sound intensity corresponding to the strongest winds at the wall of the vortex (at 659.5 minutes). The measured core pressure drop, coincident with the notch between the two maxima, was 1.1 hPa. Source data are provided as a Source Data file.

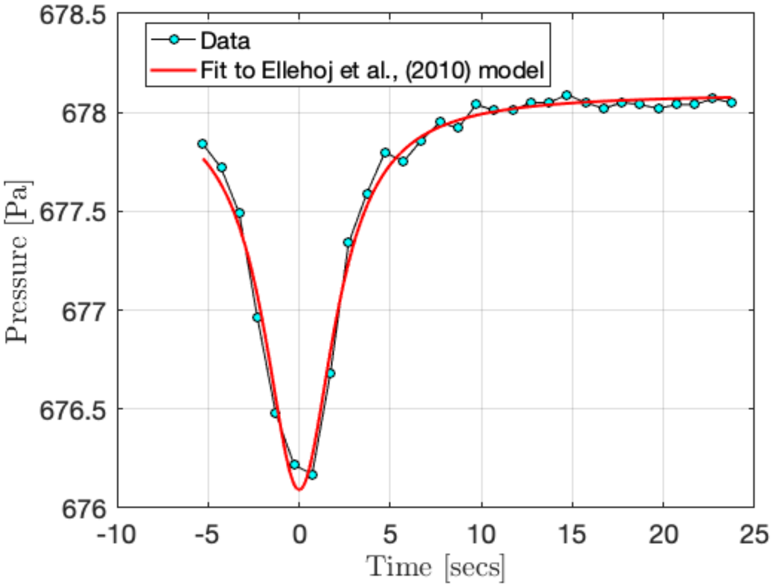
Data
Fit to Ellehoj et al., (2010) model
Pressure [Pa]
678.5
678
677.5
677
676.5
676
-10
-5
0
5
10
15
20
25
Time [secs]

(a)

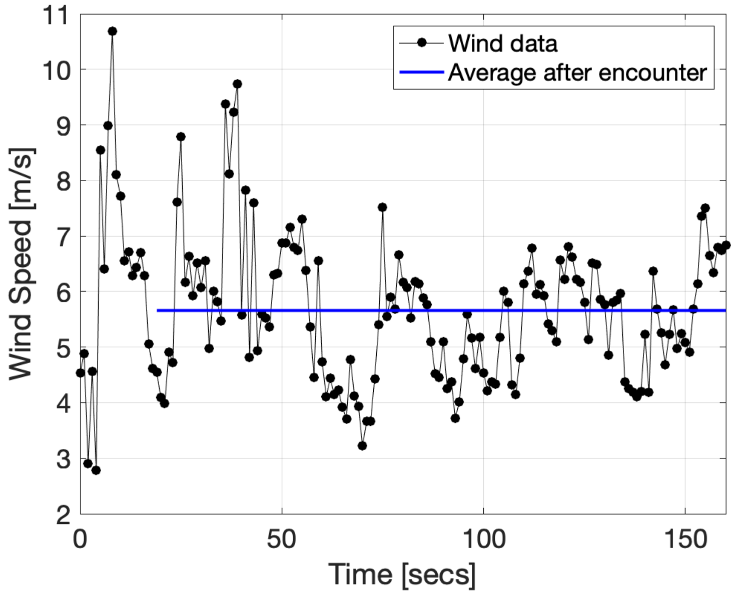
Wind data
Average after encounter
Wind Speed [m/s]
11
10
9
8
7
6
5
4
3
2
0
50
100
150
Time [secs]

(b)

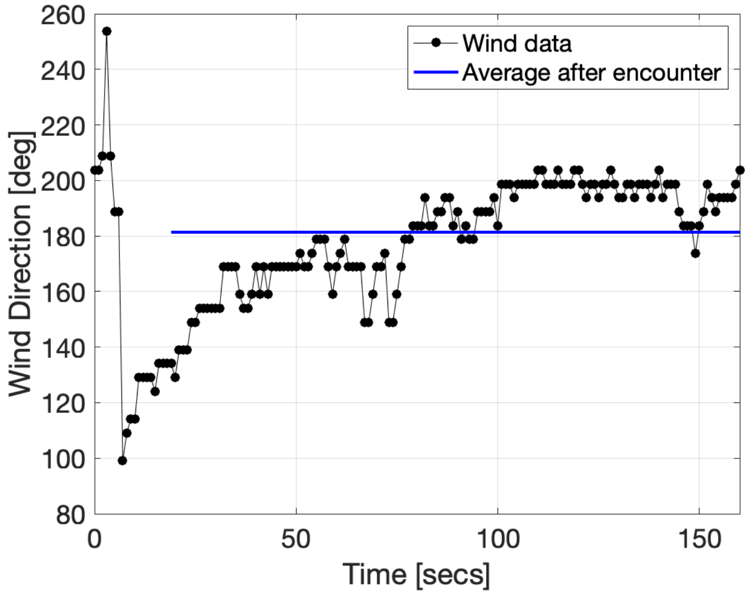
Wind data
Average after encounter
Wind Direction [deg]
260
240
220
200
180
160
140
120
100
80
0
50
100
150
Time [secs]

(c)

Supplementary Fig. 2 | Fitting the pressure data and determining the background wind speed. (a) The barometer data are indicated in light blue and the red line shows the vortex pressure model fit[2,3] to the data. The time vector is adjusted to have the pressure minimum at $t = 0$ s. The observed pressure drop is 2 Pa and the encounter duration (2 times the Full Width Half Maximum) is 9.35 s. (b) The Mars Environmental Dynamics Analyzer (MEDA) wind speed data are shown in black as a function of time from the start of the microphone recording. The background wind speed, the average wind speed from 20 to 160 s after the encounter (5.7 m/s), is shown in blue. (c) The MEDA wind direction data are shown in black as a function of time from the start of the microphone recording. The background wind direction, average wind direction from 20 to 160 s after the encounter (181°), is shown in blue. Source data are provided as a Source Data file.

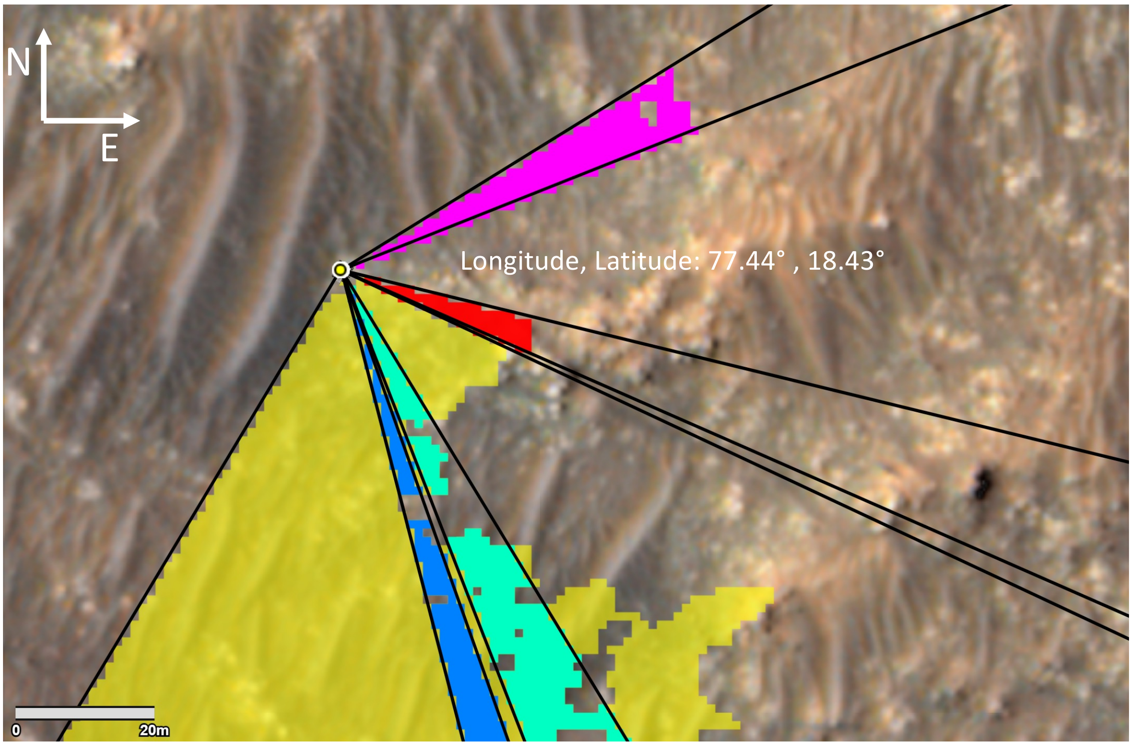

(a)

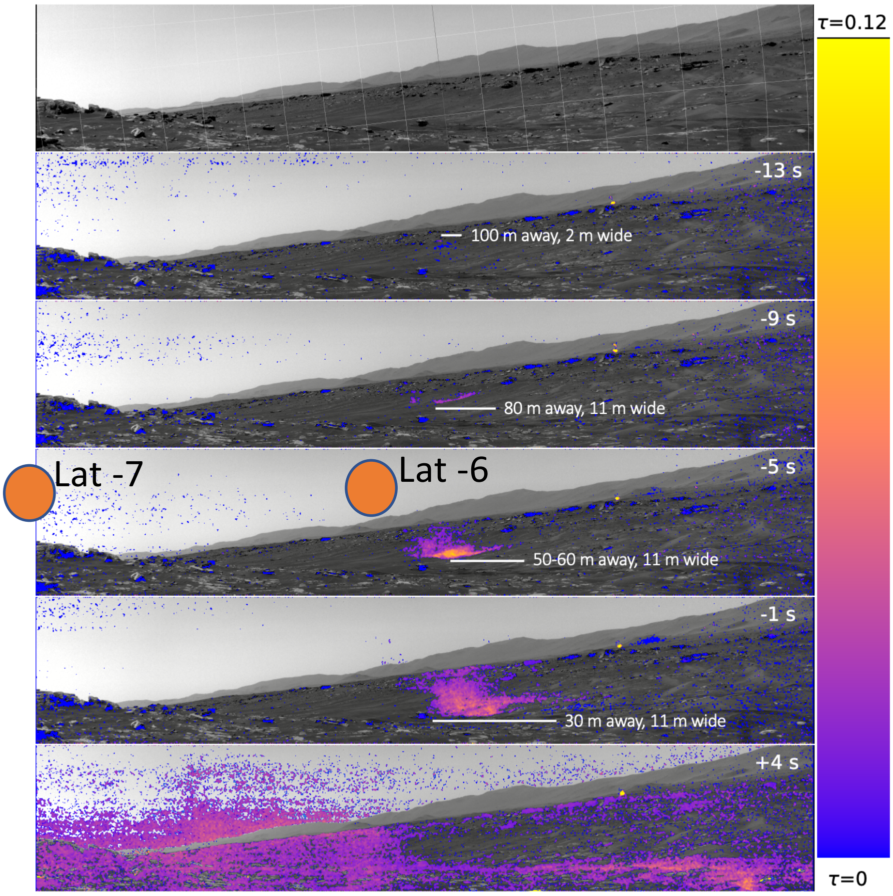

(b)

Supplementary Fig. 3 | Radiation and Dust Sensor (RDS) Field of View (FoV). (a) Context image showing the rover location on sol 215. The Navigation Camera (Navcam) viewshed is indicated in yellow (Navcam FoV => 162.99 ° ± 48 °). The field of view of the RDS sensors are also shown: Lateral (LAT) 8 (pink, FoV = 63.35 ° ± 5 °), LAT 7 (red, FoV = 108.65 ° ± 5 °) and LAT 6 (green, FoV = 153.65 ° ± 5°). LAT 5 cannot be used due to being blocked by the rover High Gain Antenna. The blue area shows the dust devil approach direction (approximately 164°). Image source: NASA/JPL-Caltech/U-Arizona[4]. (b) The same as Fig 3 of the main paper (see Fig. 3 legend for details), but indicating the pointing directions of the RDS LAT-6 and LAT-7 sensors.

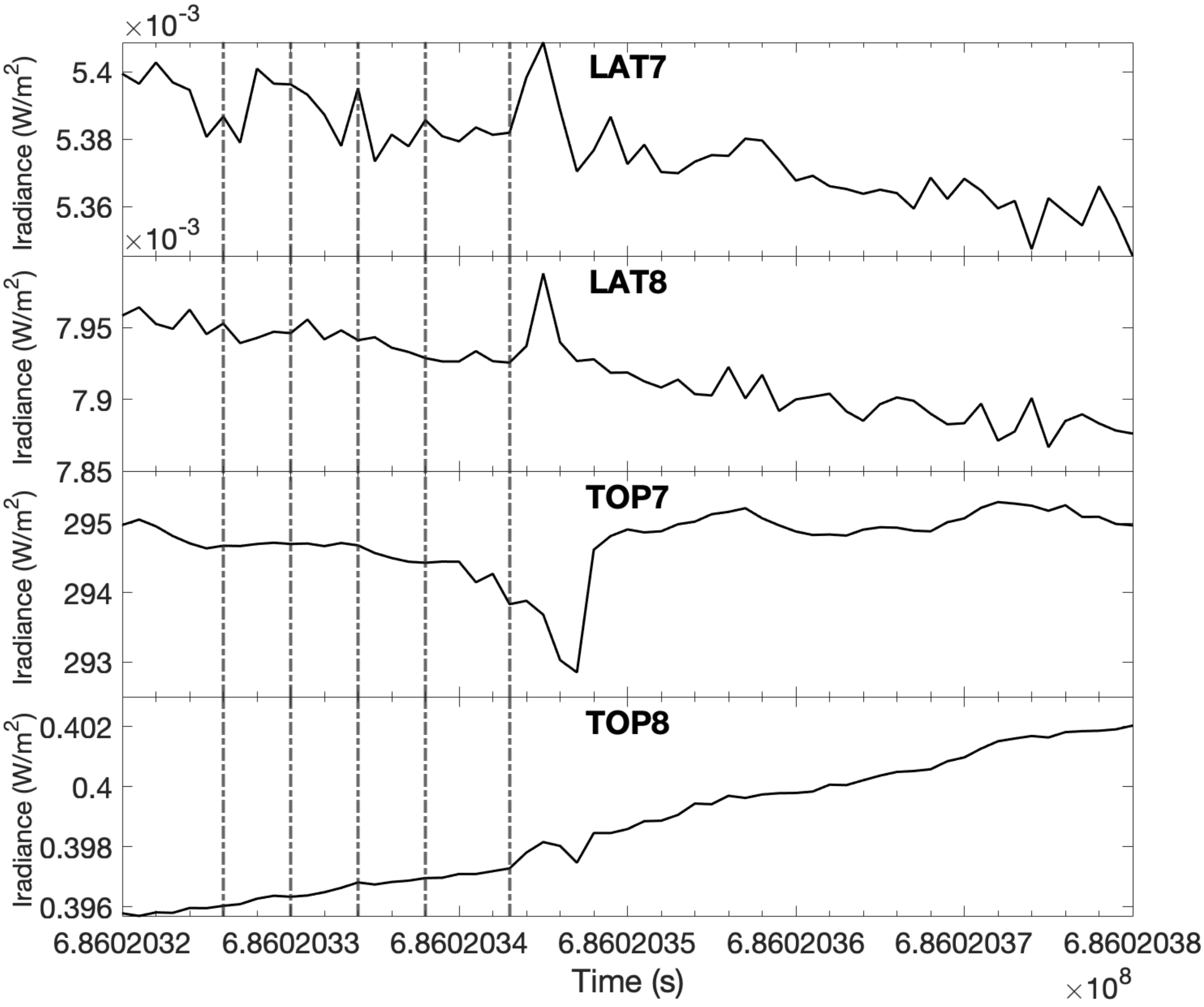
LAT7
LAT8
TOP7
TOP8
Iradiance (W/m^2)
×10^{-3}
5.4
5.38
5.36
7.95
7.9
7.85
295
294
293
0.402
0.4
0.398
0.396
6.8602032
6.8602033
6.8602034
6.8602035
6.8602036
6.8602037
6.8602038
Time (s)
×10^8

(a)

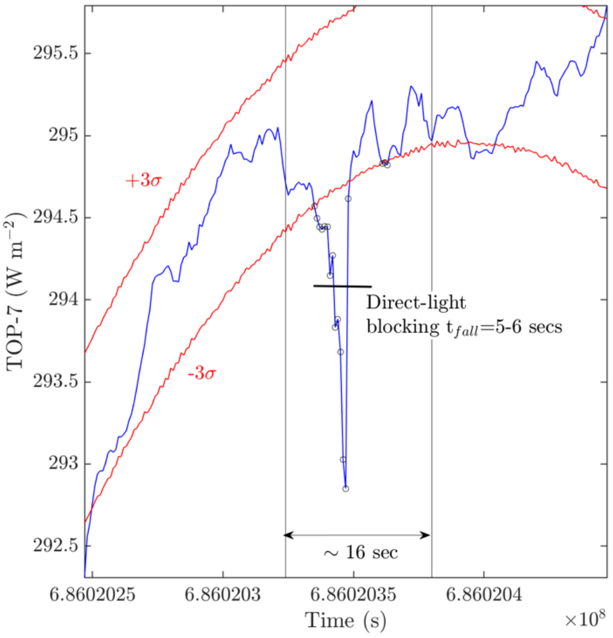
+3σ
-3σ
TOP-7 (W m^{-2})
295.5
295
294.5
294
293.5
293
292.5
Direct-light
blocking t$_{fall}$=5-6 secs
~ 16 sec
6.8602025
6.860203
6.8602035
6.860204
Time (s)
×10^8

(b)

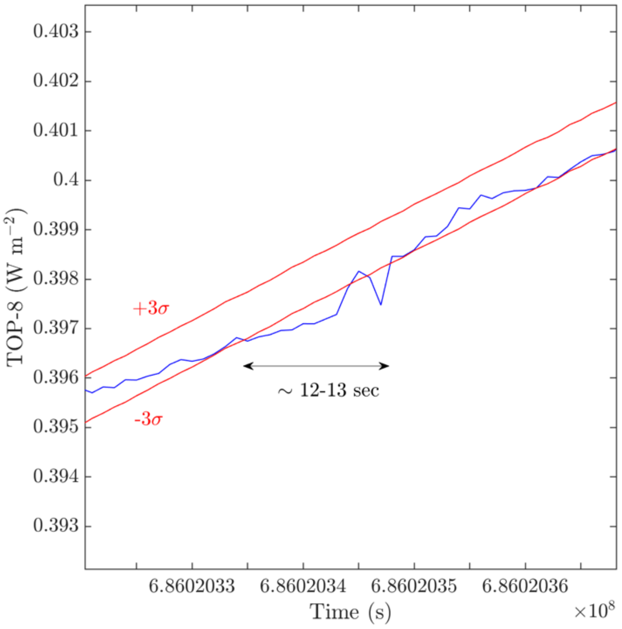
+3σ
-3σ
TOP-8 (W m^{-2})
0.403
0.402
0.401
0.4
0.399
0.398
0.397
0.396
0.395
0.394
0.393
~ 12-13 sec
6.8602033
6.8602034
6.8602035
6.8602036
Time (s)
×10^8

(c)

Supplementary Fig. 4 | Radiation and Dust Sensor (RDS) measurements. (a) Vortex signals as observed on the LAT-7, LAT-8, TOP-7 and TOP-8 RDS sensors. The LAT sensors are facing laterally and the TOP sensors are facing upwards. The vertical dotted lines indicate the times of the Navigation Camera (Navcam) frames. (b) RDS top-7 signal drop of about 16 sec produced by the presence of the dust devil, and for which a sharp decrease is observed by the blocking of the direct light (indicated with the horizontal black solid line). (c) Measured RDS signal showing the impact of the dust devil on the TOP-8 sensor (with a field of view $\pm 15°$ at zenith). These figures are shown as a function of the M2020 spacecraft clock (SCLK). The Perseverance spacecraft clock time at the start of the microphone recording is 686020339 seconds. Source data are provided as a Source Data file.

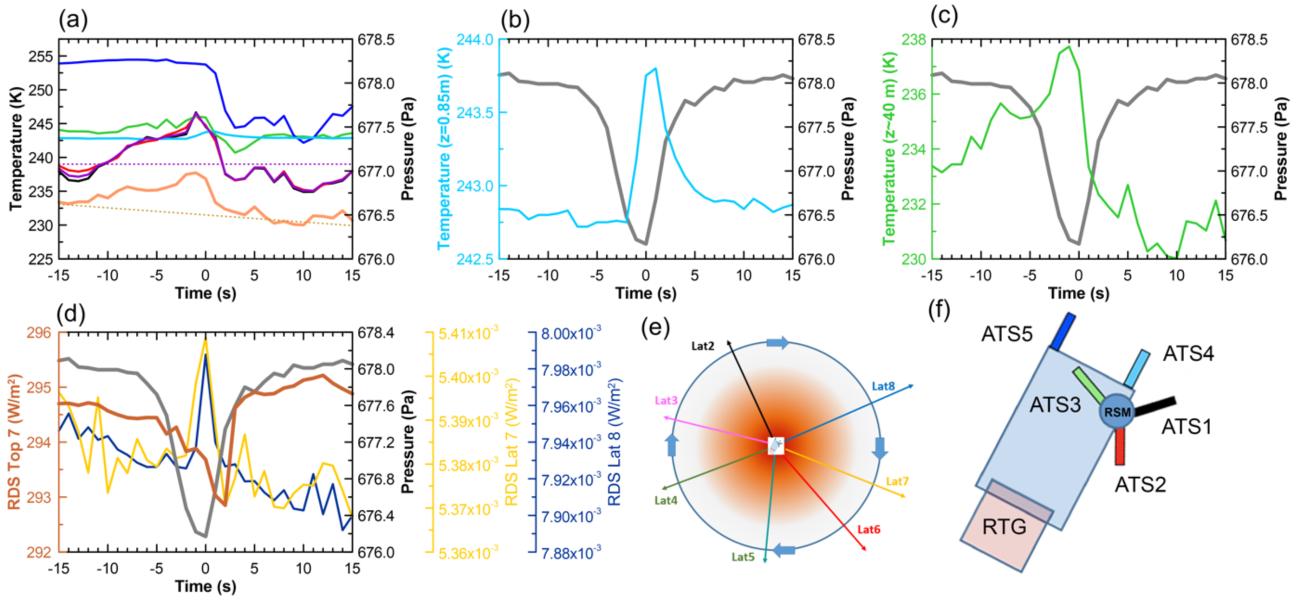

Supplementary Fig. 5 | Temperature and Radiation and Dust Sensor (RDS) data during the dust devil encounter: (a) Air temperatures and pressure. Black Red, Green are air temperature sensors (ATS) 1-3 at the Remote Sensing Mast (RSM) at height (*z*) =1.45 m. A derived temperature from these data is in magenta. Blue is ATS4 and Cyan is ATS5, both at *z*=0.85 m. ATS4 provides a better measurement of temperature. (b) ATS4 data (blue line, left axis), compared with pressure (grey line, right axis) shows temperatures increase at z=0.85 m when the vortex arrives at the location of Perseverance. The ATS5 is overheated before the vortex arrives, and cools down once the vortex passes, as the south side of the vortex brings fresh air assuming clockwise circulation. (c) Thermal Infrared Sensor (TIRS) air temperatures at a height (*z*) of approximately 40 m (green line left axis) compared with pressure. (d) RDS data with RDS Top7 and RDS Lat7 and RDS Lat 8 showing the strongest variations. (e) A schematic representation of the RDS Lat directions and Perseverance's size (black circle) compared with the estimated vortex size. (f) Orientation of the rover and positions of the different ATS in the Remote Sensing Mast (RSM) and the front of the rover. Note that ATS4 and 5 were interchanged at rover integration with respect to the original instrument description[5]. Source data are provided as a Source Data file.

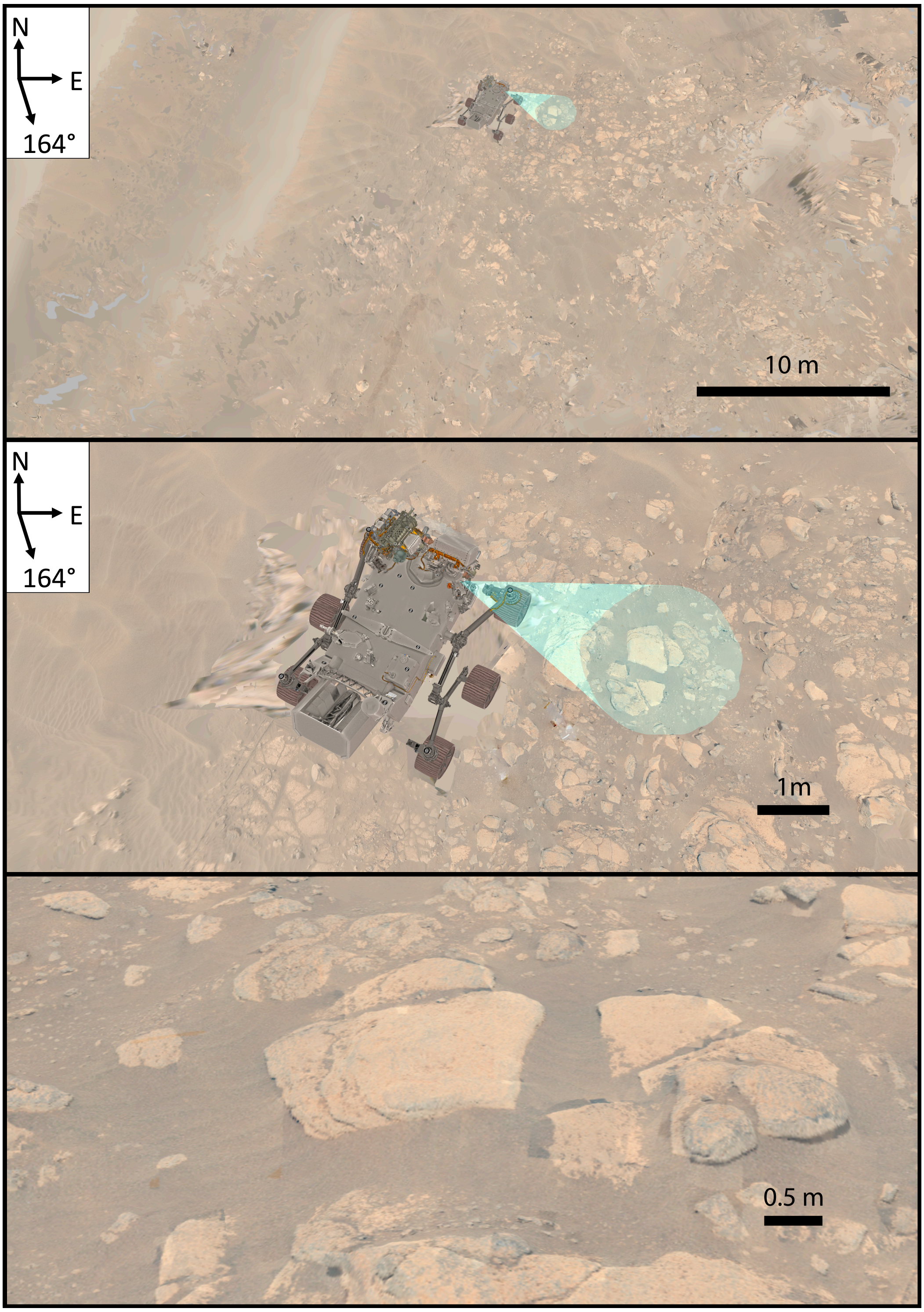

Supplementary Fig. 6 | Context images for thermal inertia measurements. (Top) A rover navigation camera (Navcam) mosaic showing the surroundings on Perseverance sol 215. (Middle) Closer view showing the approximate field of view of the Thermal Infrared Sensor (TIRS, shaded green area), used to determine the thermal inertia and albedo. (Bottom) A zoomed-in view of TIRS' field of view. The dust devil approached the rover from an azimuth of around 164° and the azimuth angle of the TIRS field of view is 102.6 degrees.

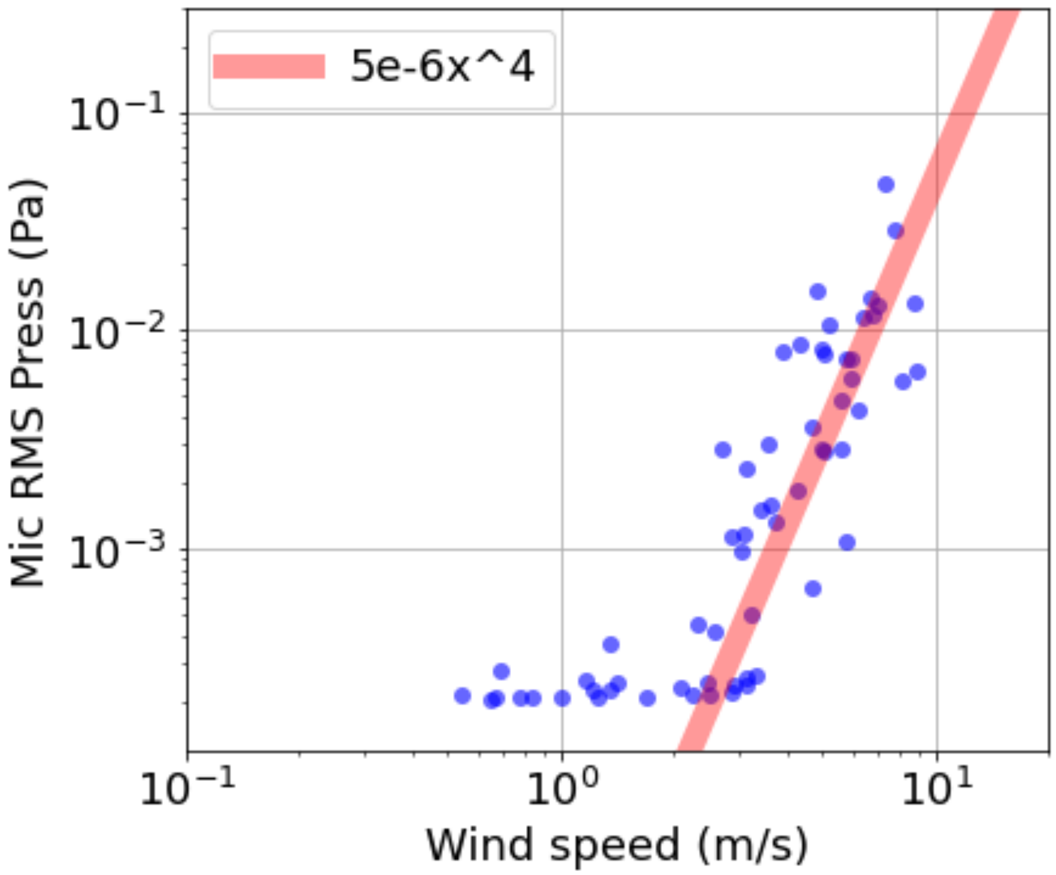

Supplementary Fig. 7 | Sensitivity of microphone to wind speed. The Root-Mean-Square (RMS) of the SuperCam microphone signal in the 20-60Hz bandwidth plotted against the mean wind speed for 62 recordings of length 167s. The wind speeds above 2 m/s can be related to the microphone signal RMS by a fourth order power law. Source data are provided as a Source Data file.

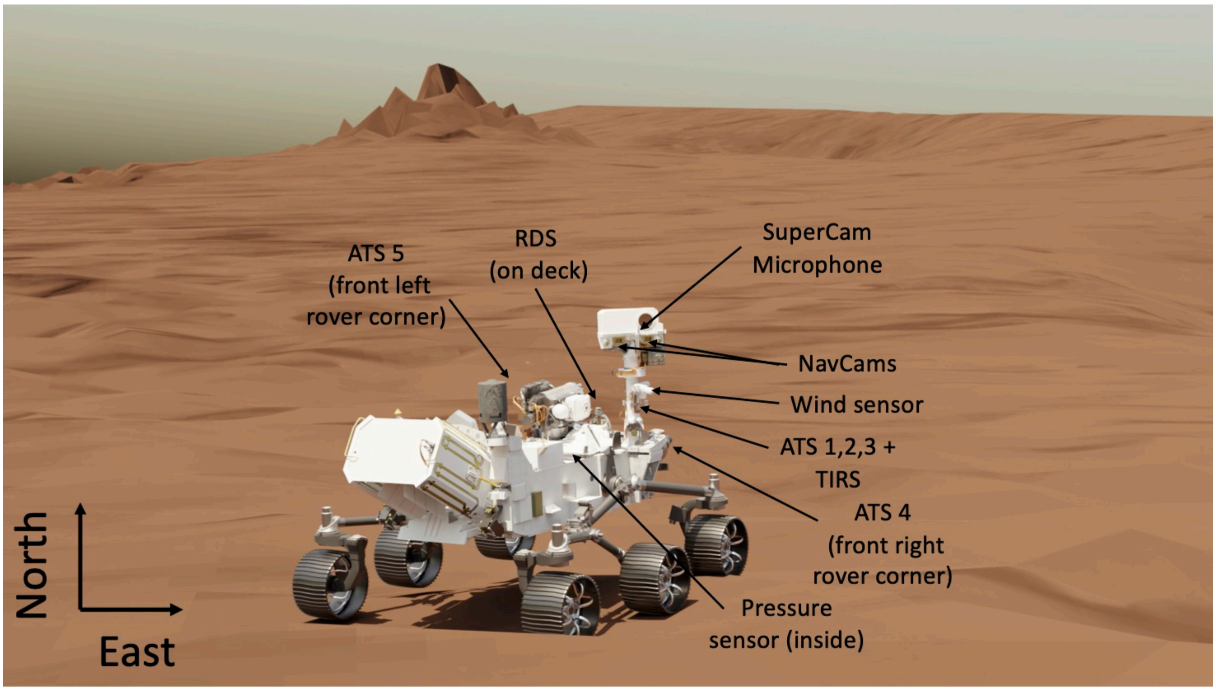

Supplementary Fig. 8 | Rover orientation on Perseverance sol 215. All of the instruments that detected the dust devil are indicated this includes the air temperature sensors (ATS), wind sensors, pressure sensor, Radiation and Dust Sensor (RDS), Thermal Infrared Sensor (TIRS), SuperCam Microphone and the Navigation Camera (Navcam). The Navcam and the SuperCam microphone are pointed in the same direction. Image credits: INTA.

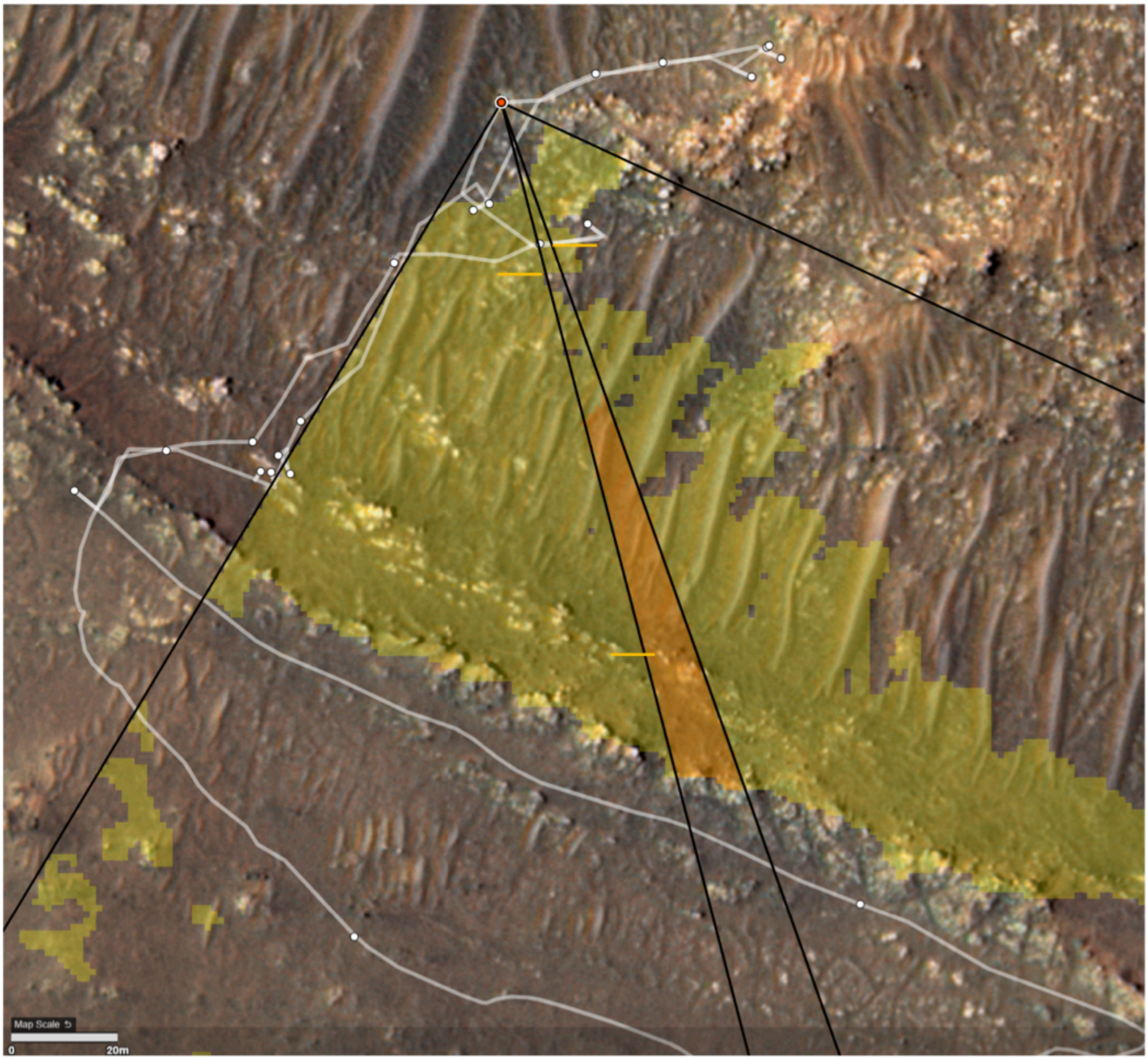

Supplementary Fig. 9 | Rover location on Perseverance sol 215. Context image showing the rover location. The Navcam Dust Devil Movie was aimed at 163° of azimuth with a viewshed indicated in yellow. The orange area is the approximate dust devil approach direction (164°). Image source: NASA/JPL-Caltech/U-Arizona[4]. Light orange lines are localized features shown here in the context image and also in the Navcam image in Supplementary Fig. 10.

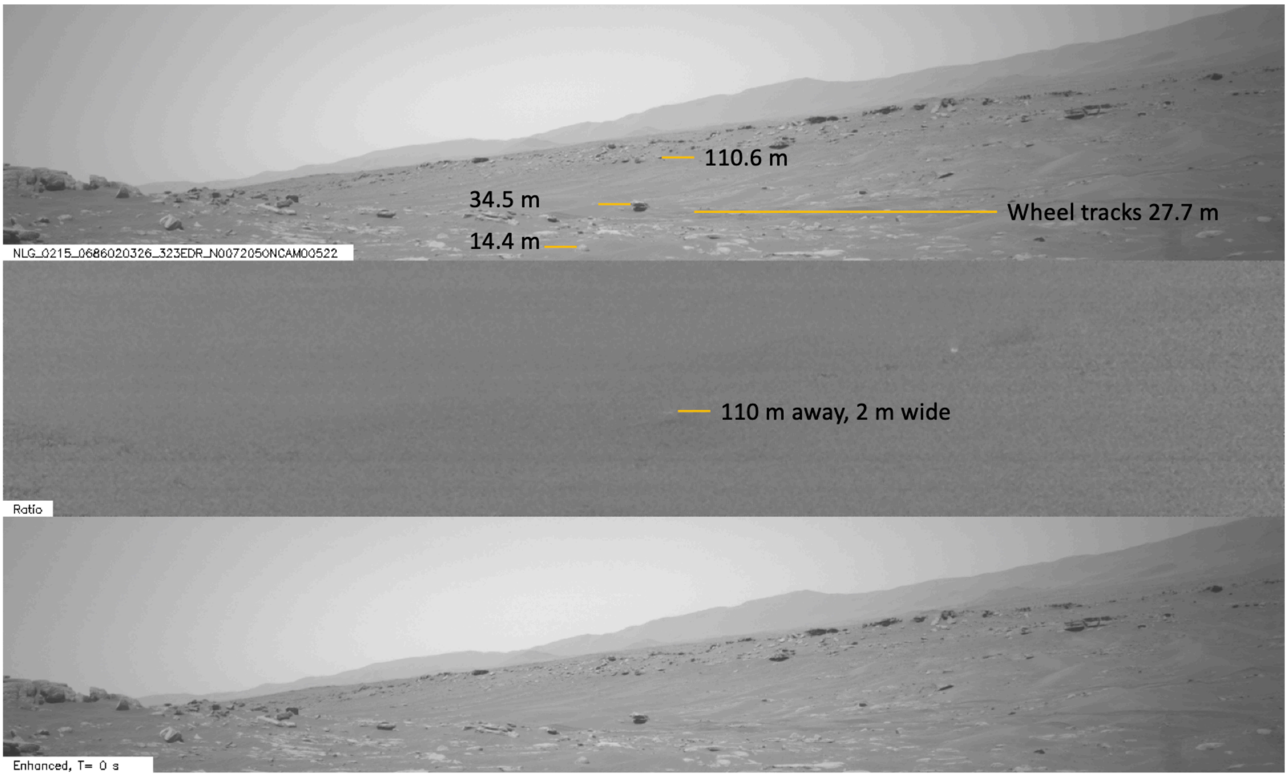

Supplementary Fig. 10 | Rover Navigation camera (Navcam) image interpretation (-13 s). (Top) The Navcam background image, (middle) the ratio of the background image and the NavCam image 13 s before the start of the SuperCam microphone recording, (bottom) the NavCam image 13 s before the start of the SuperCam microphone recording. Light orange lines in the top panel are localized features, shown in also in the context image (Supplementary Fig. 9). The orange scale bar in the middle panel shows the approximate size of the dust devil.

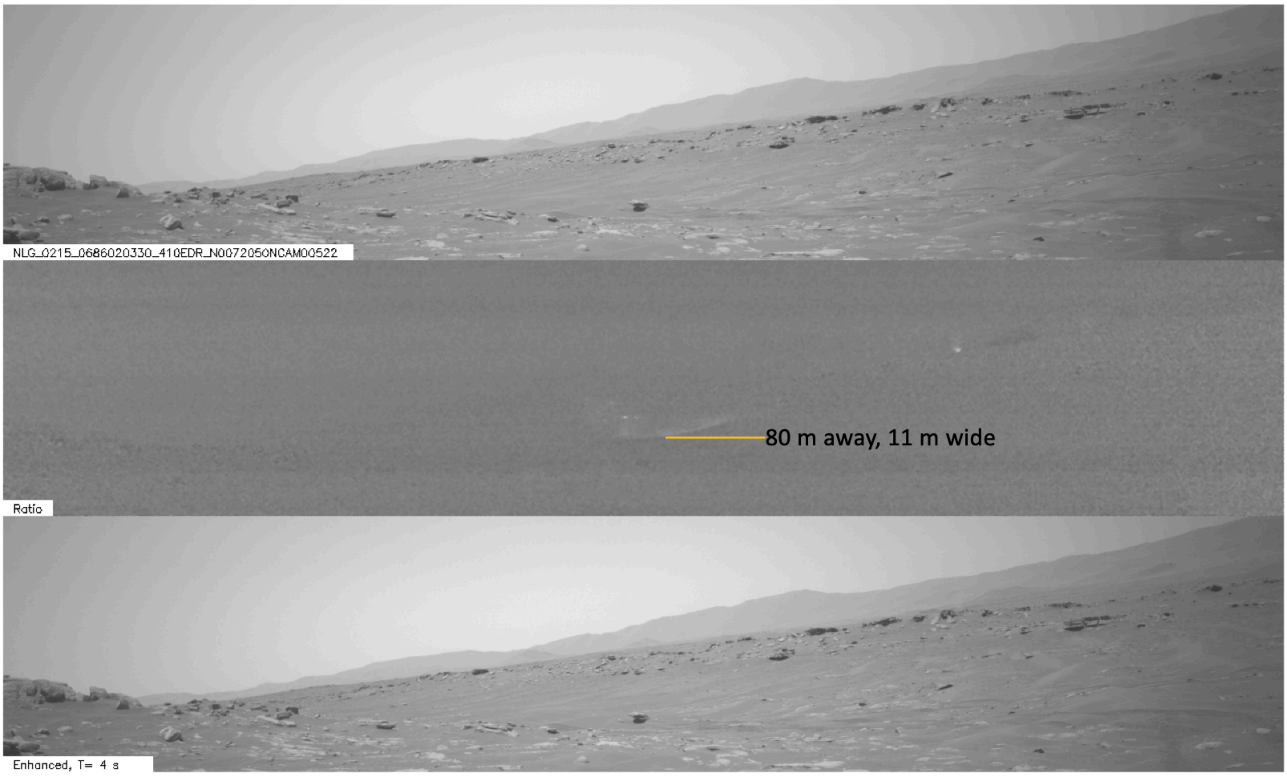

Supplementary Fig. 11 | Rover Navigation camera (Navcam) image interpretation (-9 s). (Top) The Navcam background image, (middle) the ratio of the background image and the NavCam image 9 s before the start of the SuperCam microphone recording, (bottom) the NavCam image 9 s before the start of the SuperCam microphone recording. The orange scale bar in the middle panel shows the approximate size of the dust devil.

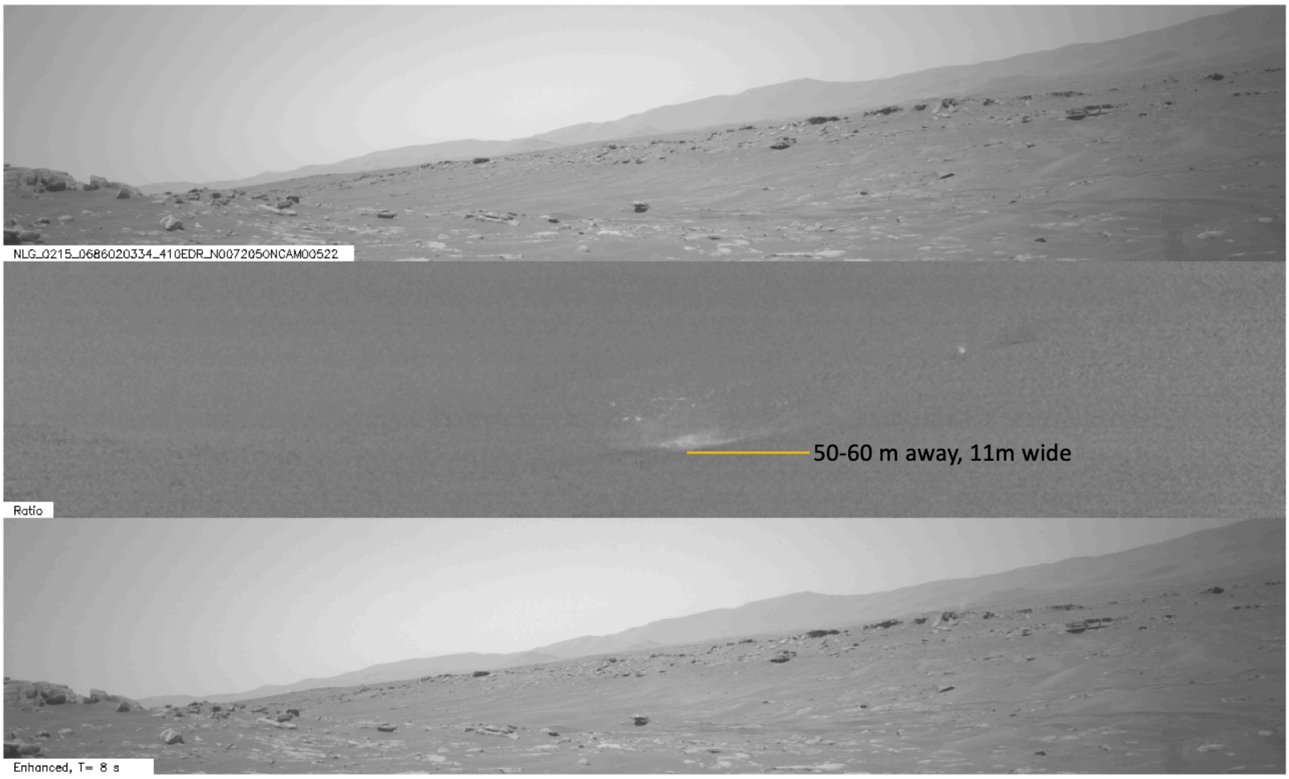

Supplementary Fig. 12 | Rover Navigation camera (Navcam) image interpretation (-5 s). (Top) The Navcam background image, (middle) the ratio of the background image and the NavCam image 5 s before the start of the SuperCam microphone recording, (bottom) the NavCam image 5 s before the start of the SuperCam microphone recording. The orange scale bar in the middle panel shows the approximate size of the dust devil.

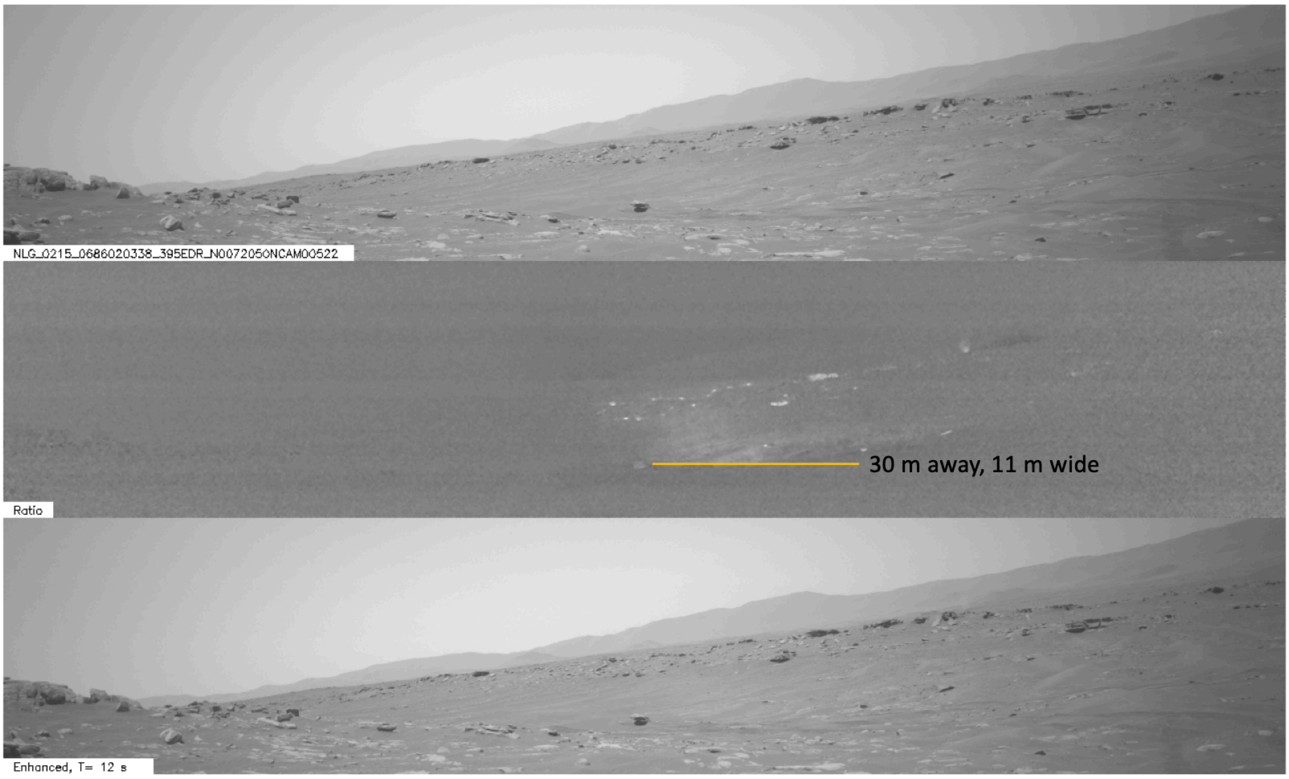

Supplementary Fig. 13 | Rover Navigation camera (Navcam) image interpretation (-1 s). (Top) The Navcam background image, (middle) the ratio of the background image and the NavCam image 1 s before the start of the SuperCam microphone recording, (bottom) the NavCam image 1 s before the start of the SuperCam microphone recording. The orange scale bar in the middle panel shows the approximate size of the dust devil.

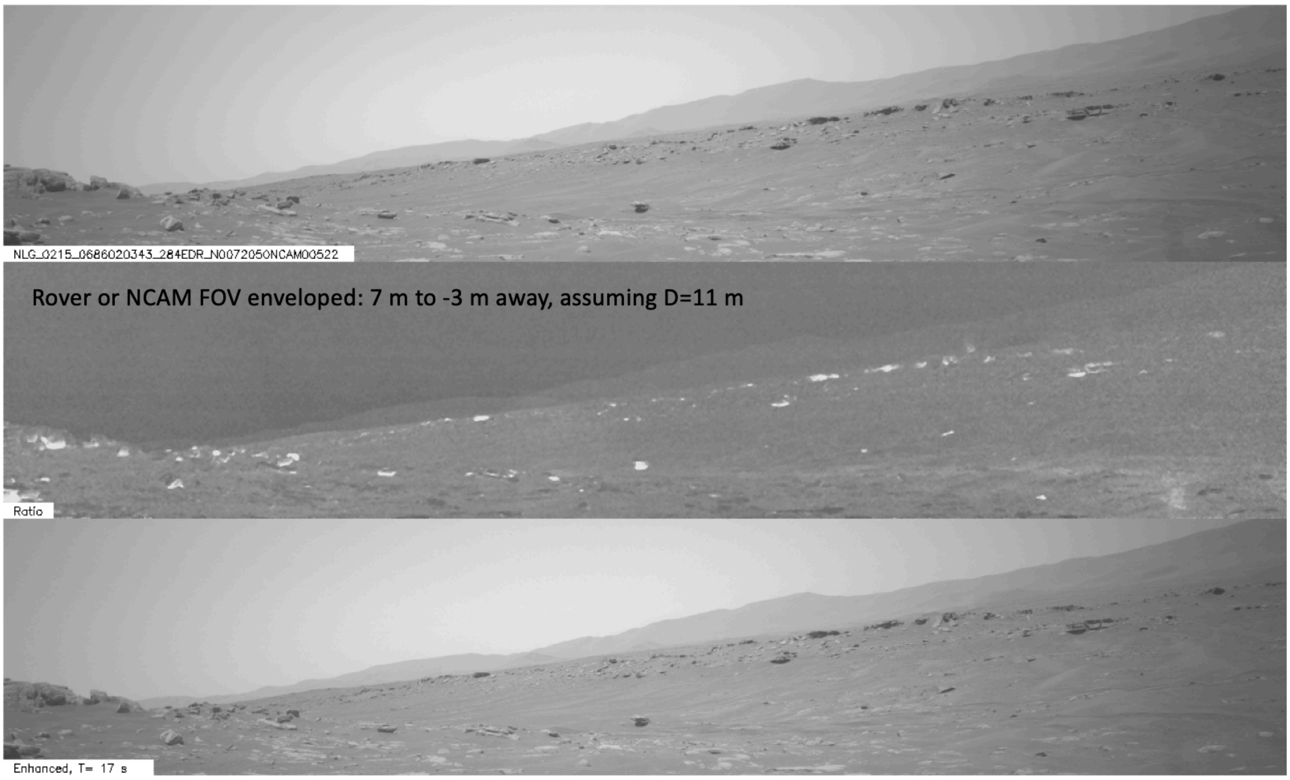

Supplementary Fig. 14 | Rover Navigation camera (Navcam) image interpretation (+4 s). (Top) The Navcam background image, (middle) the ratio of the background image and the NavCam image 4 s after the start of the SuperCam microphone recording, (bottom) the NavCam image 4 s after the start of the SuperCam microphone recording. As the rover is insight the dust devil there is no scale bar indicated the dust devil size in the middle panel.

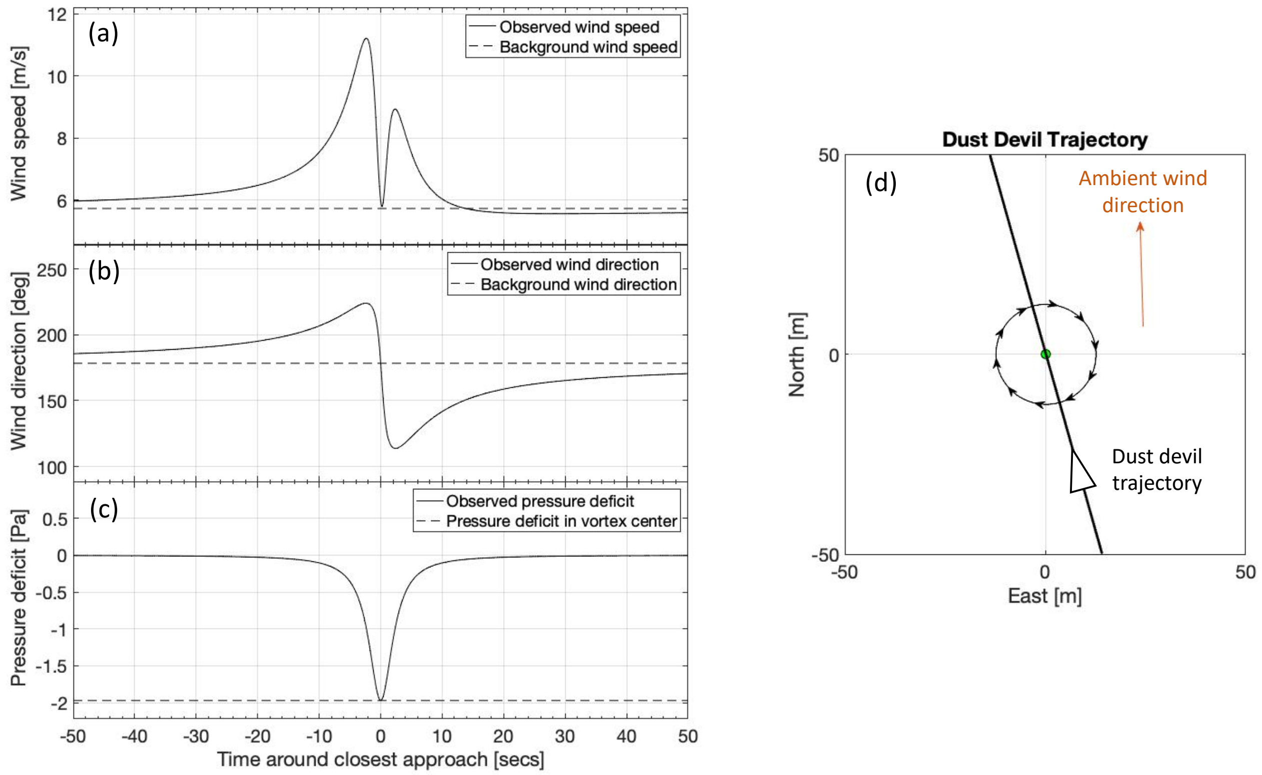

Supplementary Fig. 15 | Synthetic dust devil model. The synthetic observed wind speed (a), wind direction (b) and pressure deficit (c) for a model dust devil with the parameters given in Table 1. (d) The trajectory of the dust devil is shown in black, the circle indicates the vortex size, the small black arrows indicate the rotation direction of the vortex, the green circle is the rover position, and the orange arrow indicates the background wind direction. Source data are provided as a Source Data file.

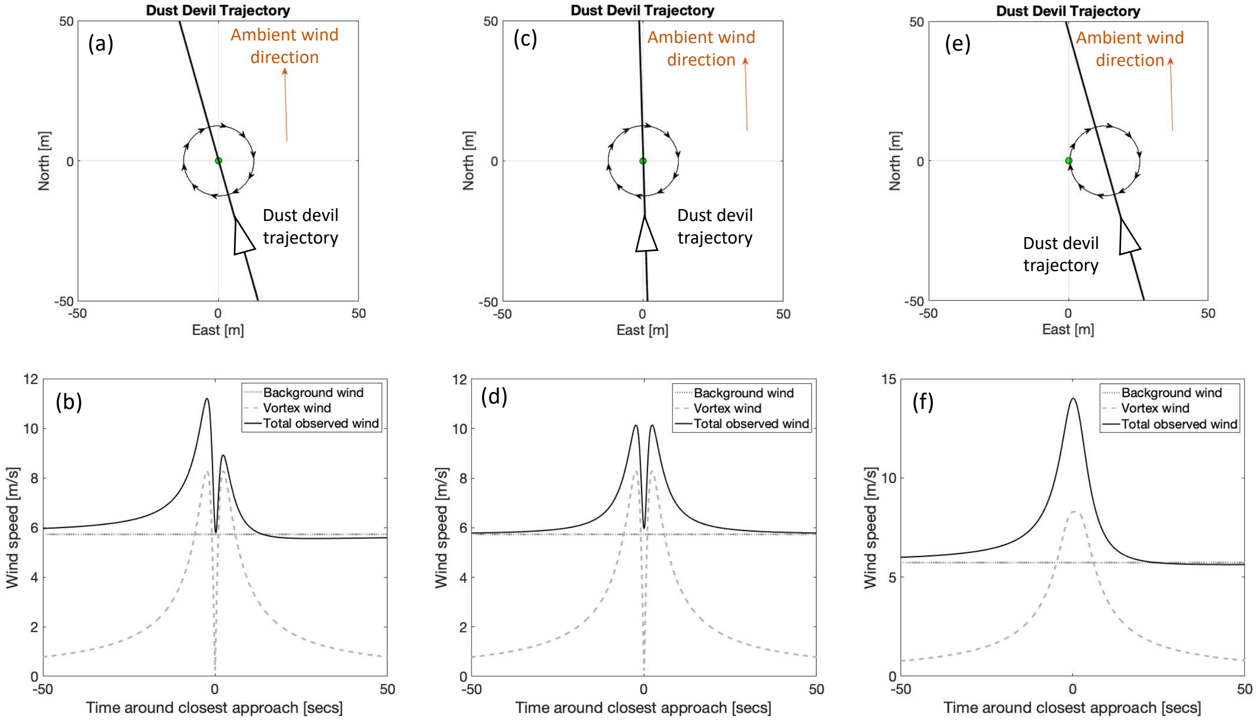

Supplementary Fig. 16 | Synthetic wind speed data for different types of dust devil encounters. The dust devil size and geometry for the encounter described in this paper are shown in (a) and the synthetic background wind (dotted line), local vortex wind (dashed line) and total observed wind (solid line) for this encounter are provided in (b). The total observed wind has an asymmetric double-lobed profile. If the dust devil trajectory direction and background wind direction were perfectly aligned (c) the resulting observed wind has a symmetric double-lobed profile (d). When the sensors encounter the wall of the dust devil, rather than the center of the dust devil (e), an increase in wind speed is observed but not double-lobe (f). In (a), (c) and (d) the trajectory of the dust devil is shown in black, the circle indicates the vortex size, the small black arrows indicate the rotation direction of the vortex, the green circle is the rover position, and the orange arrow indicates the background wind direction. Source data are provided as a Source Data file.

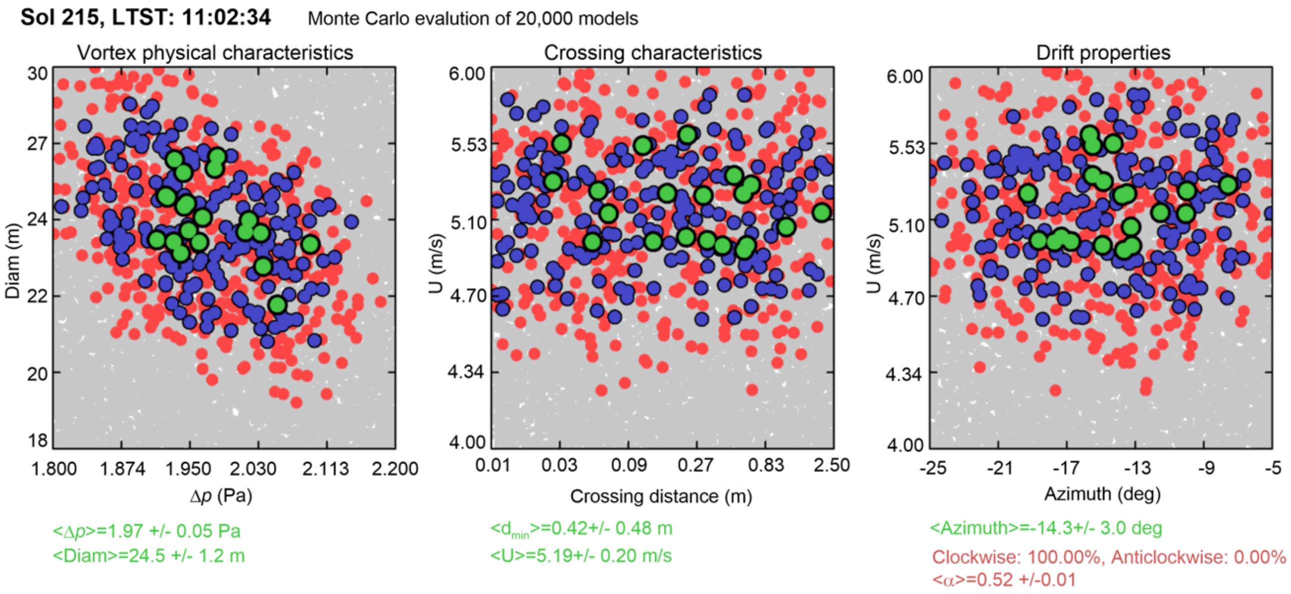

Supplementary Fig. 17 | Monte Carlo Simulation results. Results of the final stage of the Monte Carlo simulation. Of 20,000 simulations shown in grey, red shows the 600 best models, blue the 200 best models and green the 20 best models. Δp refers to the amplitude of the pressure drop at the vortex center, U is the vortex translational speed (assumed here to be equal to the background wind speed), the crossing distance (d_{min}) is the closest approach between the center of the vortex and the sensors, α is a parameter that can range from 0.5 – 1.0, where 0.5 indicates a fully cyclostrophic vortex. The azimuth in this figure indicates the direction the vortex is moving towards, rather than the value provided elsewhere in the manuscript, which is the direction the vortex is coming from. Source data are provided as a Source Data file.

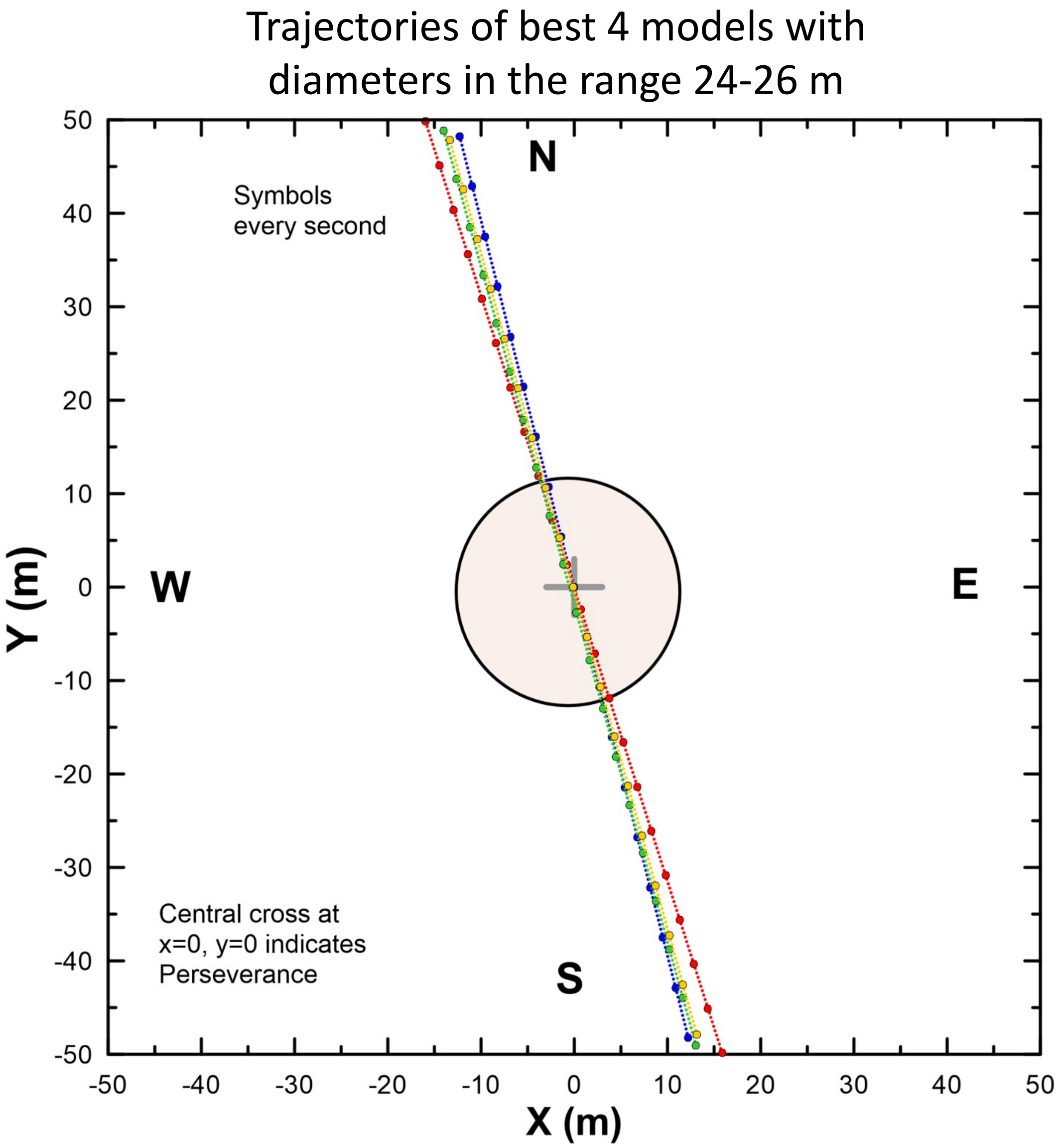

Supplementary Fig. 18 | Trajectories of the four best models from the Monte Carlo simulations. Dots are the positions of the vortex separated by time-steps of one second. Red corresponds to model number 09174, green corresponds to model number 13204, yellow corresponds to model number 19920, blue corresponds to model number 00984. Source data detailing model parameters and model results are provided as a Source Data file.

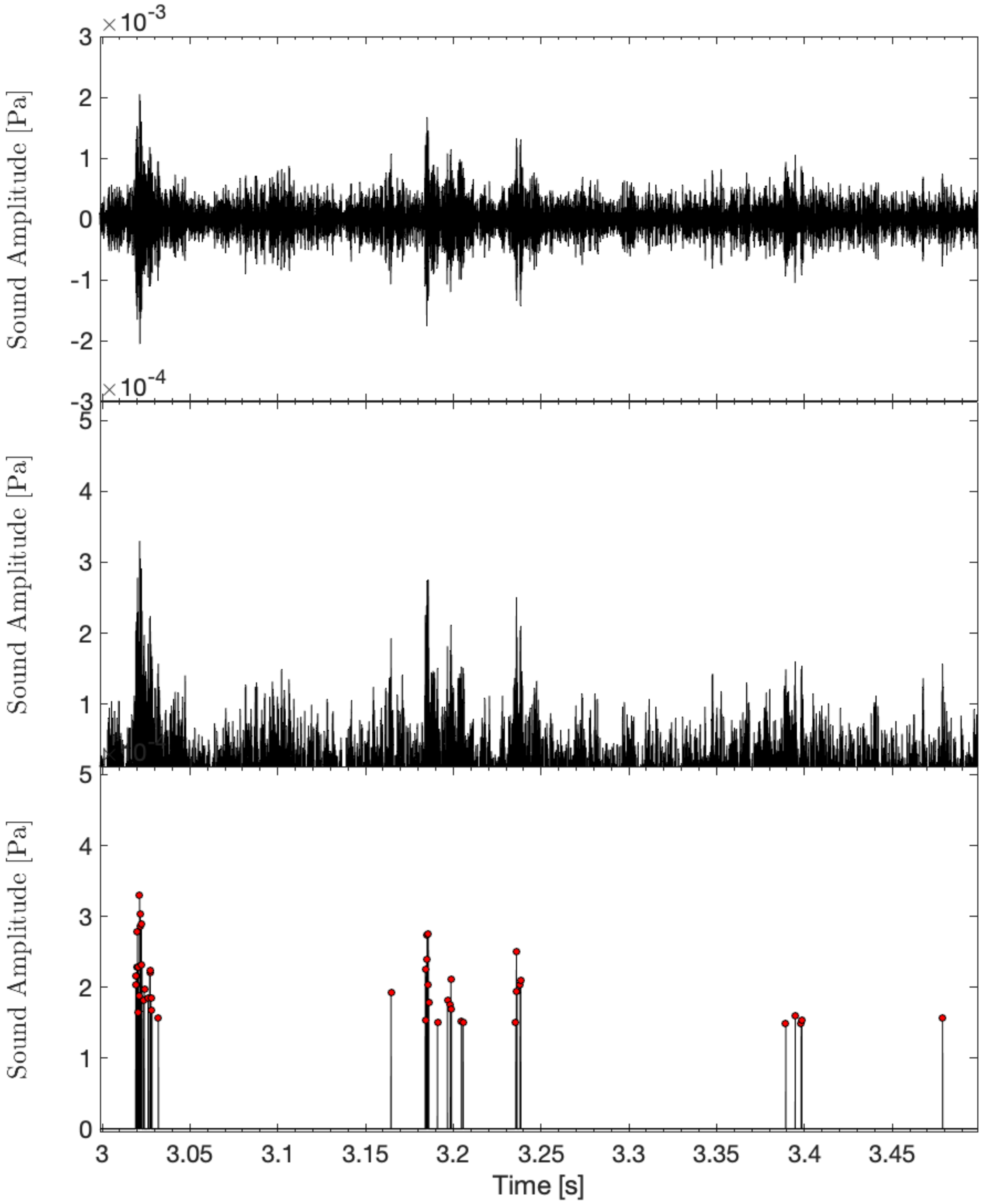

Supplementary Fig. 19 | Microphone signal processing to retrieve the frequency of grain impacts. (Top) Microphone signal (sound amplitude) filtered in the 2 – 4 kHz bandwidth during the first burst of grain impacts. (Middle) The smoothed, mean-centered signal. (Bottom) The microphone signal after all points smaller than 4 sigma have been set to zero. The red circles are the detected peaks, corresponding to grain impacts. Source data are provided as a Source Data file.

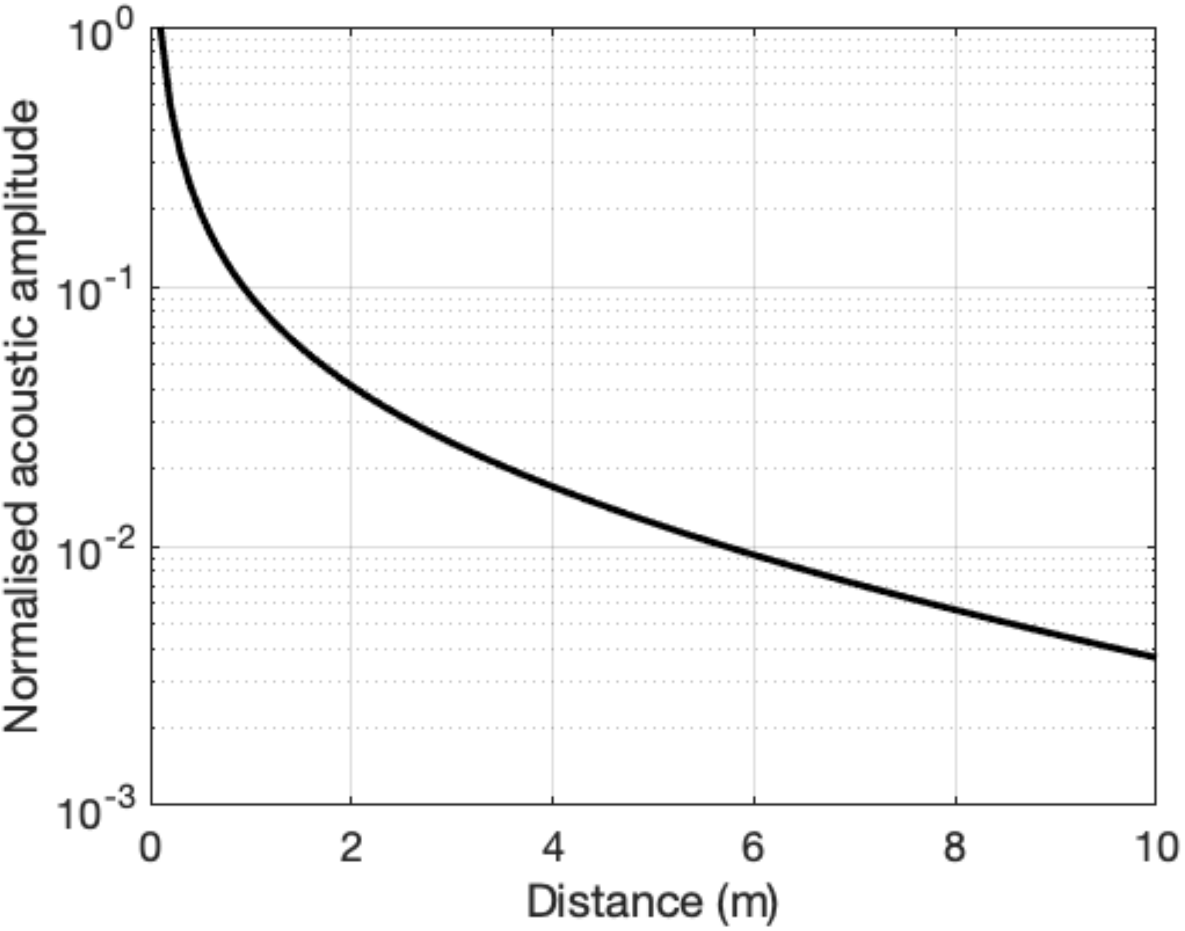

Supplementary Fig. 20 | Acoustic attenuation with distance in the 2-4 kHz bandwidth. The acoustic amplitude (normalised by the source amplitude) as a function of distance for sounds within the 2-4 kHz bandwidth assuming an attenuation coefficient, alpha, of 0.1 m^{-1} [6,7]. Source data are provided as a Source Data file.

# - Supplementary References–

[1] Murdoch, N. et al. Predicting signatures of dust devils recorded by the SuperCam microphone. *in 52nd Lunar and Planetary Science Conference, Held Virtually*. No 2583. (LPI, 2021).

[2]Ellehøj, M. D. et al. Convective vortices and dust devils at the Phoenix Mars mission landing site. *J. Geophys. Res*, 115, E00E16 (2010).

[3]Murdoch, N. et al. Constraining martian regolith and vortex parameters from combined seismic and meteorological measurements. *J. Geophys. Res.: Planets*, e2020JE006410 (2021).

[4]Calef, F. J., T. Soliman, and H. E. Abarca. Multi-Mission Geographic Information System: Updates and Science Operations Status." In *51st Annual Lunar and Planetary Science Conference*. No. 2326. (LPI 2020)

[5]Rodriguez-Manfredi, J. A. et al. The Mars environmental dynamics analyzer, MEDA. A suite of environmental sensors for the Mars 2020 mission. *Space Sci. Rev*, 48 (2021).

[6]Maurice, S. et al. In situ recording of Mars soundscape. *Nature* 605, 653–658 (2022).

[7]Bass, H. E. & Chambers, J. P. Absorption of sound in the Martian atmosphere. *J. Acoustical Soc. Am*. 109, 3069–3071 (2001).
